# Supplementary material for: Resonance energy transfer sensitises and monitors in situ switching of LOV2-based optogenetic actuators
Source: Nat Commun. 2020 Oct 9;11:5107. doi: 10.1038/s41467-020-18816-8 (PMC7547724; doi:10.1038/s41467-020-18816-8)
Supplement: Supplementary file 1 — Supplementary Information [file 41467_2020_18816_MOESM1_ESM.pdf]

Supplementary information

Resonance energy transfer sensitises and monitors *in situ* switching of LOV2-based optogenetic actuators

Li-Li Li[1,2,3], Florence M Klein[1], Lorenzo Li Greci[1], Arkadiusz Popinigis[1,4], Florian Freudenberg[5], Michael J Courtney[1,2,6*]

[1]Neuronal Signalling Lab, Turku Bioscience Centre, University of Turku and Åbo Academy University, Biocity, Turku, Finland

[2]Turku Screening Unit, Biocity, Turku, Finland

[3]Current Address: Metabolic Research Laboratories, Wellcome-MRC Institute of Metabolic Science, University of Cambridge, Cambridge, UK

[4]Current Address: BLIRT S.A., Trzy Lipy 3/1.38, 80-172 Gdansk – Poland.

[5]Department of Psychiatry, Psychosomatic Medicine and Psychotherapy, University Hospital, Goethe University, Frankfurt, Germany

[6]Corresponding Author, email: michael.courtney@bioscience.fi

A

$$E = \frac{1}{1 + \left(\frac{r}{R_0}\right)^6}$$

$$w_F = \frac{1}{\tau_{A^*}} \left(\frac{R_0}{r}\right)^6$$

$$w_F = \frac{9\kappa^2 c^4}{8\pi\tau_{A^*} n^4 R^6} \int F_A(\omega)\, \sigma_B(\omega) \frac{d\omega}{\omega^4}$$

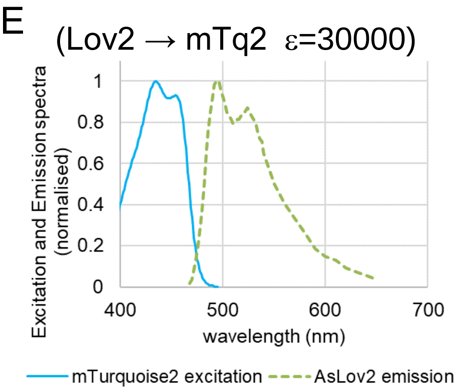

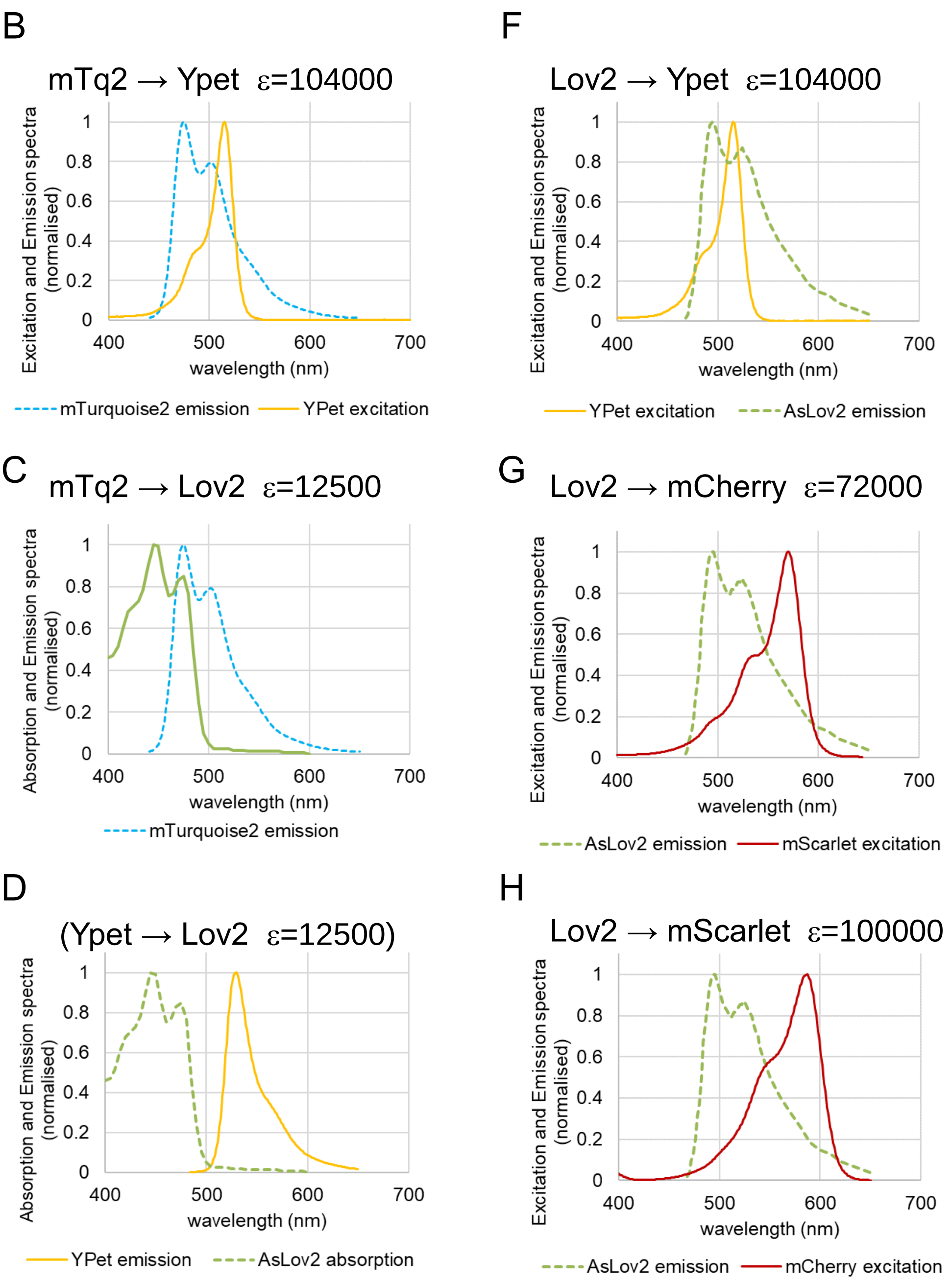

Supplementary Figure 1: Spectral overlap of potential resonance energy transfer donors and acceptors for LOV2-bound FMN.

(A). Efficiency of resonance energy transfer is strongly related to distance. The actual rate of energy transfer (w) depends also on donor activated state lifetime (τ_{A*}), spectral overlap, extinction coefficient, and relative dipole angles[1].

(B-H) Normalised spectral data and acceptor extinction coefficients for fluorescent proteins were obtained from FPbase[2] and for *As*LOV2, dark-state absorption and excited-state emission spectra from Gauden et al. (2004)[3] and dark-state ε from Diensthuber et al. (2014)[4]. Spectral overlap of the classical mTurquoise2 (mTq2)/Ypet FRET pair (B) is similar to overlap with ground-state FMN-bound *As*LOV2 absorption spectrum, LOV-445 (C). Overlaps of Ypet emission with LOV-445 absorption (D), or of (E) S1 excited-state LOV* emission with mTurquoise2 excitation are minimal. Overlap between Ypet excitation and LOV* emission spectra is substantial (F). The LOV* emission spectrum also overlaps with the excitation spectra of red proteins mCherry and mScarlet (G-H). ε in B-H refers to extinction coefficient (in $M^{-1}cm^{-2}$) of the second fluorophore named (candidate RET acceptor).

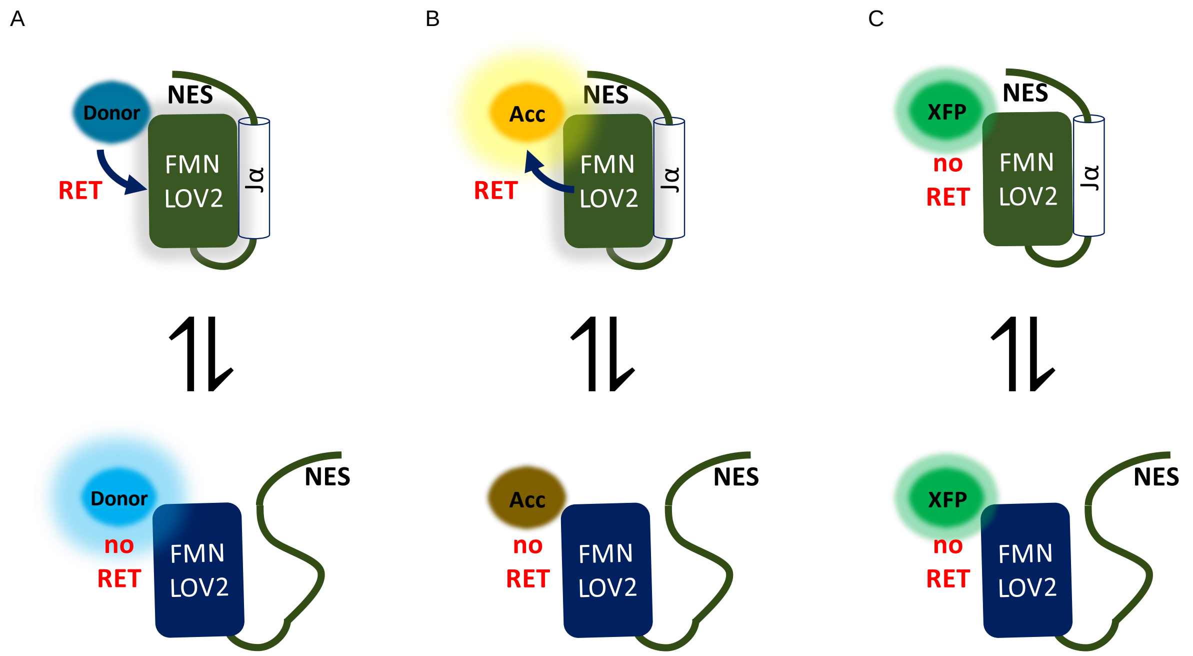

Supplementary Figure 2: Three scenarios are envisaged for the impact of fluorescent protein fusions on LOV2 switching.

(A) Sensitisation by cyan proteins. In cases where the fluorescent protein can act as a donor, the protein can absorb additional photons and transfer energy to FMN-bound LOV2, which can alternatively absorb photons directly. This would increase the rate of formation of adduct state. As RET efficiency depends on spectral overlap, donor activation lifetime and acceptor extinction coefficient[1] (Supplementary Figure 1A), we predicted mTurquoise2, an excellent RET donor for yellow proteins[5], may act as a RET donor for LOV-445 and sensitise the switching of LOV2 by blue light.

(B) Suppression by yellow and red proteins: Emission spectra of LOV-445 and mTurquoise2 are similar, thus yellow protein Ypet might act as a RET acceptor ("Acc") from LOV* as it does for cyan proteins, reducing FMN triplet state (LOV^T) formation, impeding light-induced LOV-390 generation and subsequent conformational changes. The red protein mCherry is typically used in LOV2-based optogenetic fusions, but here we use mScarlet[6] due to higher long-term solubility than mCherry (Supplementary Figure 3). Both can act as acceptors for mTurquoise as a RET donor[6] and their excitation spectra overlap considerably with LOV2-bound FMN emission spectrum, suggesting these fusion tags may also act as RET acceptors and impede FMN-dependent optogenetic actuation.

(C) No effect: an alternative possibility is that conditions may be unsuitable for RET, and fluorescent protein fusion might have no effect. Possible causes include an unfavourable angle factor k when using a highly rigid linker, or inter-fluorophore distance well above R_0 (typically 5nm – depending on parameters in the formula in Supplementary Figure 1A). However, XFPs and LOV2 are very small domains and inter-fluorophore distance is unlikely to be so limiting. A further option is that the first two options might co-occur as they do in homo-FRET, and whether the outcome is sensitisation or inhibition becomes hard to predict.

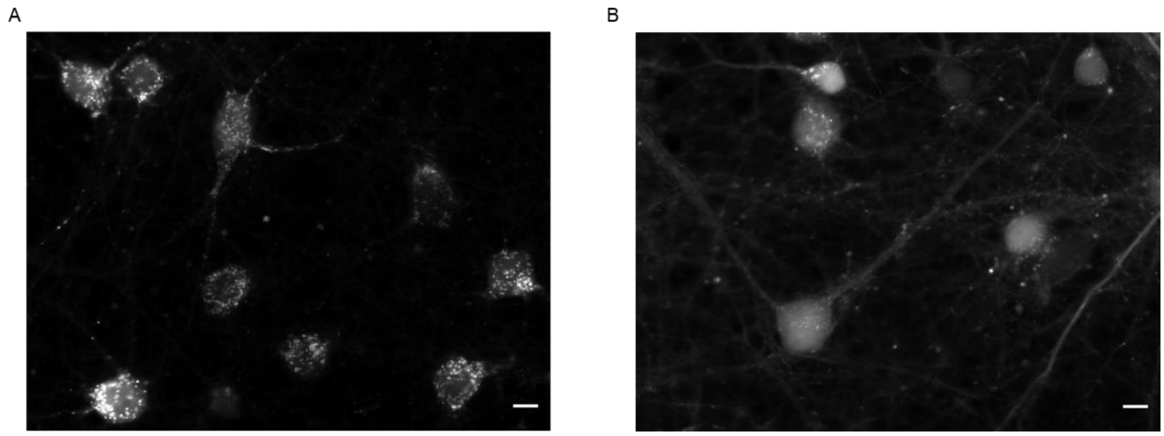

Supplementary Figure 3: Replacement of mCherry with mScarlet reduces long term aggregation

(A) Long-term expression of mCherry fused to a pEGFP-C1 type multiple cloning site (mCherry-C1) in rat hippocampal neurons leads to the formation of bright aggregates with little fluorescence remaining outside the aggregates; (B) the corresponding conditions for the improved fluorescent protein mScarlet[6] in exactly the same fusion construct i.e. mScarlet-C1, greatly limits the formation of these aggregates. Although the fusion peptide (in this case the MCS) influences the extent of aggregation, based on this observation we avoid using mCherry in this work. Images were taken 9 days after addition of corresponding AAV particles to 3 DIV neuron cultures (scale bar: 10µm). Image acquisition and processing settings were identical in both cases – acquired through a 40x NA0.95 air objective at 0.1 second exposure with 555/28nm excitation filter, 645/75nm emission filter, processed with ImageJ rolling ball setting 500 pixels and contrast adjusted linearly between pixel values 0 and 750. (n= 30 cells for mCherry and 20 cells for mScarlet.)

A

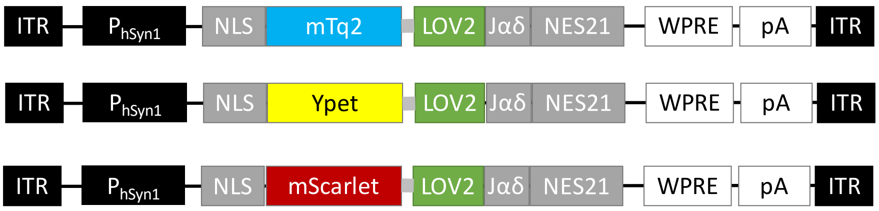

B

```
                               -----XFP--- --linker--- ----MCS----- |->  L404-LOV2
Ypet-optoNES               218-HMVLLEFLTAA GITEGMNELYK SGLRSRAQASNS LATTLERIEKNFVITDPRLPDNPIIFASDSFLQL
mTq2-optoNES               218-HMVLLEFVTAA GITLGMDELYK SGLRSRAQASNS LATTLERIEKNFVITDPRLPDNPIIFASDSFLQL
mScarlet-optoNES           212-VVEQYERSEGR HSTGGMDELYK SGLRSRAQASNS LATTLERIEKNFVITDPRLPDNPIIFASDSFLQL
LEXY (Niopek et al., 2016) 215-IVEQYERAEGR HSTGGMDELYK GGSGGSGG---S LATTLERIEKNFVITDPRLPDNPIIFASDSFLQL
                                    .        . .. .... ............ ..................................
```

Supplementary Figure 4: AAV expression construct structure and optoNES designs used

(A) Organisation of functional components in the AAV constructs generated for the experiments shown in Fig. 1.

(B) Alignments across the fusion boundary of XFP-optoNES constructs used in Fig. 1A-E, in comparison with the originally mCherry-LEXY[7], on which the constructs were based. The “δ” of Jαδ indicates that part of the Jα is replaced by NES21 in these constructs. Shown are the end of the fluorescent protein (sequence alignments based on structural data and alignments), a conventional 11aa C-terminal linker, 12 common amino acids from the “EGFP-C1” multiple cloning site (replaced in the original construct by a triple GGS sequence) and AsLOV2 starting at residue leucine 404. This amounts to a 23 amino acid linker between the fluorescent protein and the LOV2 domain and 20 in the original construct. Identities are indicated with a dot. The small difference between our design and the original was implemented for convenience in cloning when comparing different fusion proteins, it is not our intention to make any direct comparison to the original construct. For this reason we refer to the constructs we use as optoNES not LEXY. To limit background export rate and enhance dynamic range in response to activation, we include the NLS sequence N-terminal to the fluorescent protein as described in the original design[7].

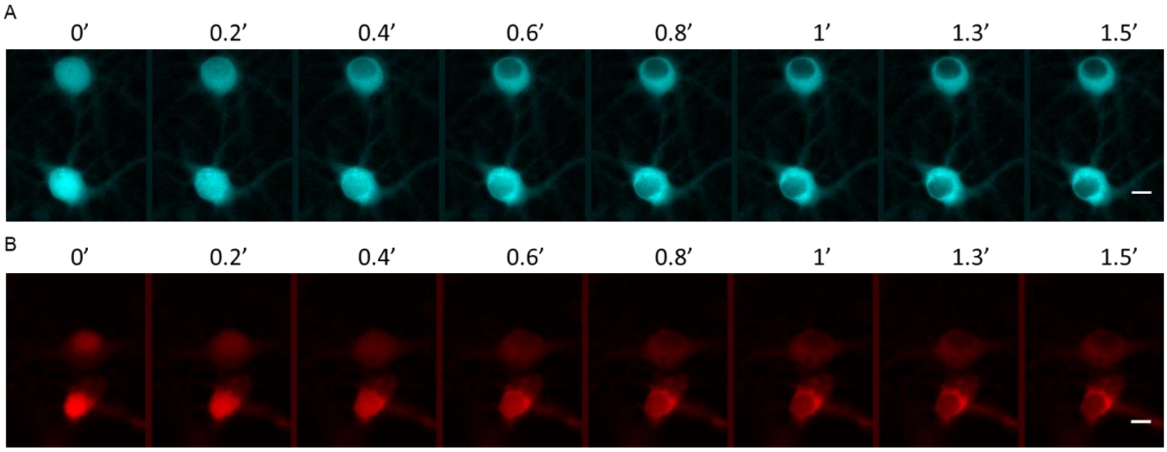

Supplementary Figure 5: Time-series showing fluorescent protein fusions of optoNES to light.

Blue-light (333µmol.m^{-2} photons at 438nm for each frame) induces export of (A) mTurquoise2-tagged and (B) mScarlet-tagged optogenetic nuclear export construct XFP-optoNES (Supplementary Figure 4) expressed separately in different rat hippocampal neurons (7DIV, 40x NA0.95 air objective; scalebar: 10µm). Images shown were acquired every ~13 seconds as indicated. To ensure consistent illumination, images in mTq2, mScarlet, YPET and miRFP670 channels were acquired regardless of the actual fluorescent protein expressed. This is achieved by exposing the samples to 500nm, 555nm and 635nm light before each 438nm exposure. Images were background subtracted and normalised to correct for intensity fluctuations. The full 40 frame image movies are in Supplementary Movies 1-6. (n=4 independent experiments representative of at least 8 wells at 40x).

Note that the level of translocation at any time point depends on the light dose (fixed here), the time-point (indicated), the light-dependence of the construct (the parameter we wished to determine), the maximal response size (i.e. dynamic range) and the initial translocation level. The changes over time in these image series shown at a single light dose here are dominated by the maximal response and initial states over the light sensitivities and a faster rate may result from the larger maximal response even where the sensitivity is lower. But the focus of the present study is sensitivity, which is determined from a series of such experiments at different light doses. Supplementary Figure 9 shows that these constructs exhibit different maximal responses.

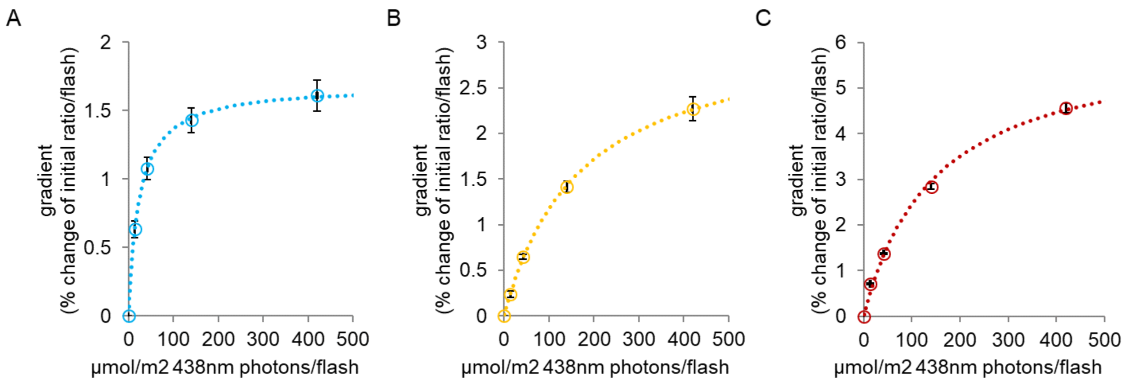

Supplementary Figure 6: Non-normalised versions of data shown in Fig. 1E.

(A) mTq2-optoNES; (B) Ypet-optoNES; (C) mScarlet-optoNES; Mean ± SEM are shown (n=6 wells). Source data are provided as a Source Data file.

A

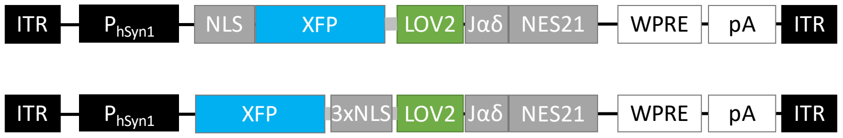

B

```
                          -----XFP--- ---LINKER------- |--------3xNLS---------| --MCS-- |->L404-LOV2
NLS-mTq2-LOV2NES21     218-HMVLLEFVTAA GITLGMDELYKSGLRS                          RAQASNS LATTLERIEKNFVI
mTq2-3xNLS-LOV2NES21   218-HMVLLEFVTAA GITLGMDELYKSGLRS DPKKKRKVDPKKKRKVDPKKKRRS RAQASNS LATTLERIEKNFVI
                           ........... ................                          ....... ..............
```

Supplementary Figure 7: Design of optoNES constructs incorporating a 24 residue spacer between the fluorescent protein and the LOV2 domain

(A) Comparison of the optoNES AAV constructs with the original design (upper, NLS at N-terminus, no additional spacer between XFP and LOV2) and the modified design (lower), in which the N-terminal NLS is replaced with a 3-copy version (24 amino acids) that is used as a spacer between XFP and LOV2. This modification limits cytoplasmic levels and increases the nuclear/cytoplasmic ratio at baseline (Supplementary Table 1; Supplementary Movies 1-6). Independently of this, it also allows the effect of increased XFP-LOV2 distance on the photon-dependence of the response to be determined (Fig 2; Table 1; Supplementary Table 1).

(B) Alignments of mTq2-optoNES constructs without spacer (NLS at N-terminus of XFP) or with a 24 amino acid spacer (3x NLS as spacer between XFP and LOV2) to limit resonance energy transfer between XFP and LOV2 domains. Identities are indicated with a dot.

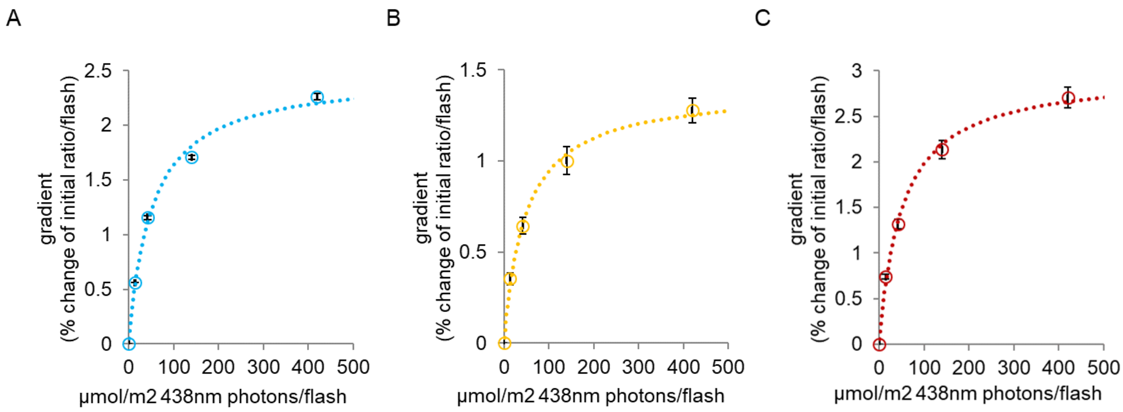

Supplementary Figure 8: Non-normalised versions of data shown in Fig. 1F.

(A) mTq2-spacer-optoNES; (B) Ypet-spacer-optoNES; (C) mScarlet-spacer-optoNES; Mean ± SEM are shown (n=6 wells). Source data are provided as a Source Data file.

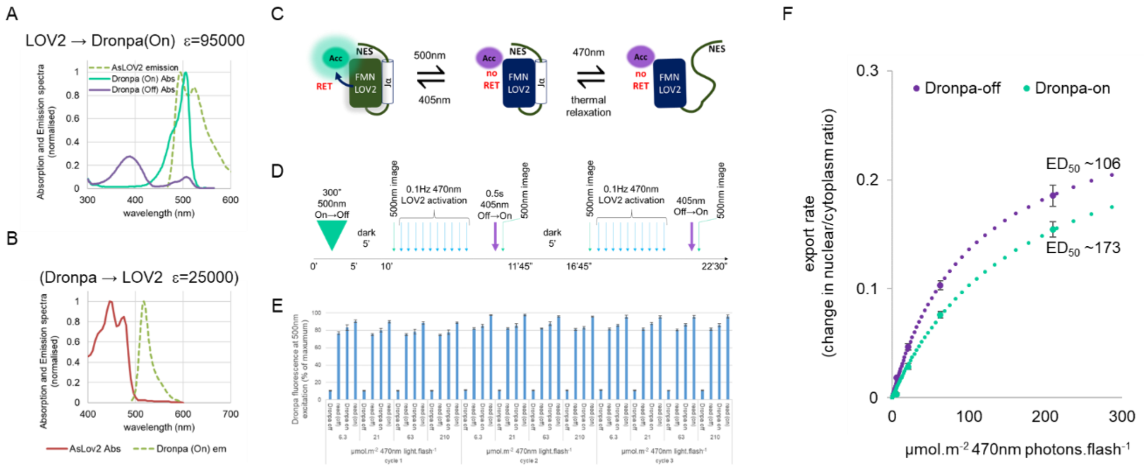

Supplementary Figure 9: LOV2 sensitivity modulation by RET with optoNES fused to a reversibly photoswitchable protein

(A) Absorbance spectra[10] suggest Dronpa-On should be an efficient RET acceptor for LOV2*.

(B) In contrast RET from exited-state Dronpa to LOV2 should be negligible.

(C) LOV2 activation is expected to be more efficient in Dronpa-Off state than Dronpa-On state.

(D) Single illumination cycle for measuring actuation sensitivity of Dronpa-optoNES. It starts with Dronpa off-switching (500nm, 0.231mol.m-2 in 300s), 5 min dark recovery period prior to optoNES testing, a 500nm image to confirm Dronpa was off, sensitivity was tested with a LOV2 activation train - 10 flashes at 0.1Hz at 6.3, 21, 63 or 210µmol.m-2.flash-1; 470nm light was used to avoid switching Dronpa. Dronpa-on switching (405nm 2.3mmol.m-2 in 0.5s) is followed immediately by a 500nm image to determine optoNES localisation after the LOV2 activation train. This was followed by an identical repeat of all but the initial off-switching step, to ensure LOV2 activation and quantification conditions were constant within the cycle, with the exception of the Dronpa state. Thus four low intensity 500nm images (77µmol.m-2) were taken per cycle, showing i) Dronpa is switched off, ii) the optoNES response in the Dronpa-off condition, ii) Dronpa on-state, confirming 5 min is sufficient time for recovery to baseline optoNES localisation, iv) the optoNES response in the Dronpa-on condition. Locations of nuclei (miRFP670-3xNLS) were captured during the first 30s of every dark period. For clarity this is omitted from the scheme, as 635nm is not absorbed by Dronpa or AsLOV2.

(E) Dronpa-fluorescence (at 500nm) indicate ~90% shutdown of Dronpa fluorescence at each cycle whereas subsequent images show similar intensity (mean ± SEM, n=6 are shown). Three cycles, (4 different 470nm LOV2-activation photon doses each), were carried out (total 12 cycles as in D).

(F) Nucleocytoplasmic ratio changes are shown for 4 different 470nm photon doses, fitted to single-site kinetics as in Fig. 1. This shows that Dronpa-optoNES in the Dronpa-off state is more sensitive to light than when in the Dronpa-on state. This provides further evidence that RET is responsible for modulation of optoNES sensitivity, and shows that sensitivity of a blue-light responsive actuator can be dynamically modified *in situ* (The best-fit values ± S.E., n=6 wells). Source data are provided as a Source Data file.

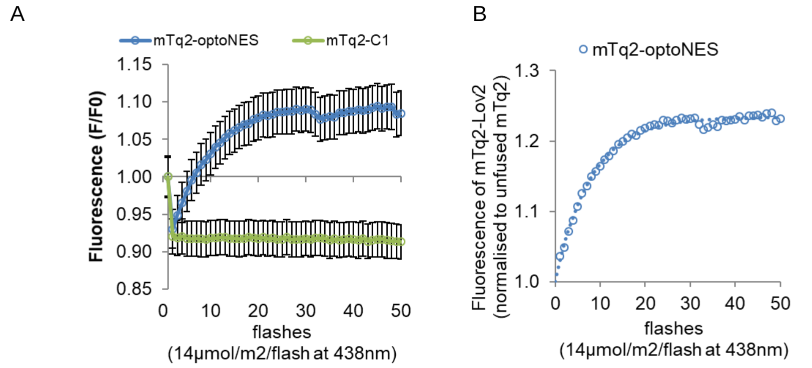

C

$A_{0(quenching)}$ = 23%
$\tau_{dequenching}$ = 7.9 flashes
Apparent rate constant ~110 µmol/m2

Supplementary Figure 10: LOV2-fusion dequench response correction by normalisation against LOV2-free fluorescent protein

(A) Even well-optimised fluorescent proteins exhibit intrinsic partial rapid photobleaching[8,9]; we observed this in particular with mTurquoise2 (green circles and line). It was essential to normalise quench/dequench data to the properties of LOV2-free XFP controls under identical conditions, which records the small initial quench observed with unfused XFP (mTurquoise2 in this case, encoded on construct mTq2-C1). In all quench/de-quenching data presented in this report the data is normalised to parallel sample wells of cells expressing unfused XFP (mTurquoise2, Ypet, mScarlet) as controls. Data from a single well is shown here as an example as mean of all ROIs (95 and 59 in this case) from the field ± SEM.

(B) After the trace in A is normalised to the unfused XFP control, curve fitting indicated it was clear that the protein was initially quenched by ~20%. Data from a single well is shown here as an example. (C) Curve fit parameters for this single well dataset are shown, A0 is the magnitude of the dequench curve as a % of fitted initial value pre-illumination, τ is the time constant of the curve in number of flashes which, when multiplied by the photon dose per flash provides an apparent rate constant.

Source data are provided as a Source Data file.

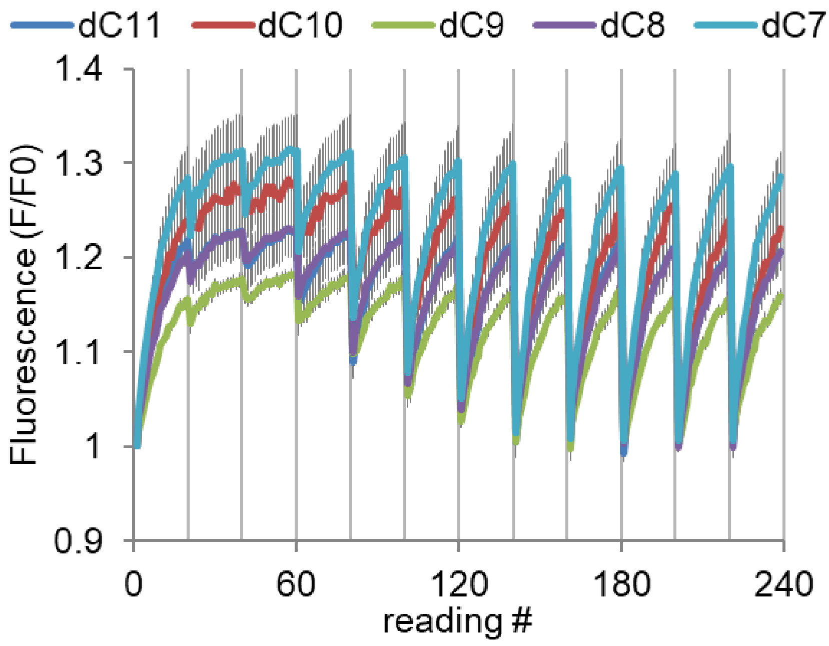

Supplementary Figure 11: The data of Fig. 5C is shown here with error bars, as means ± SEM (n=3 wells).

Source data are provided as a Source Data file.

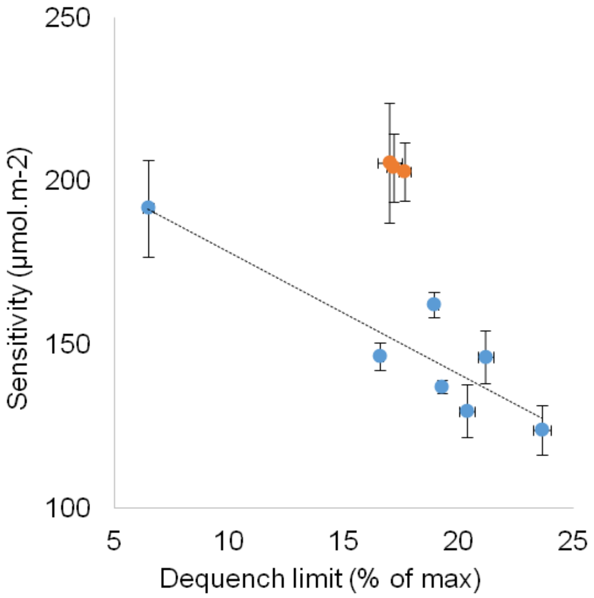

Supplementary Figure 12: Sensitivity-dequench relationship.

Plot of best-fit activation sensitivity and dequench limits for constructs mTq2dC7-408LOV2-NES21, mTq2dC8-408LOV2-NES21, mTq2dC9-408LOV2-NES21 and mTq2dC10-408LOV2-NES21, mTq2-optoNES and mTq2-spacer-optoNES (all in blue) ± S.E. (n=6 wells for wild-type, n=3 wells for variants), with the linear regression from Fig. 5F shown. Here, corresponding values for optoJNKi, optop38i3 and optop38i5 are also shown, in orange. The latter constructs have a different design that keeps the Jα sequence intact, therefore increased stability is expected, which could explain the reduced sensitivity (higher µmol.m^{-2} values). The data shown are from Table 1 and the source data are provided as a Source Data file.

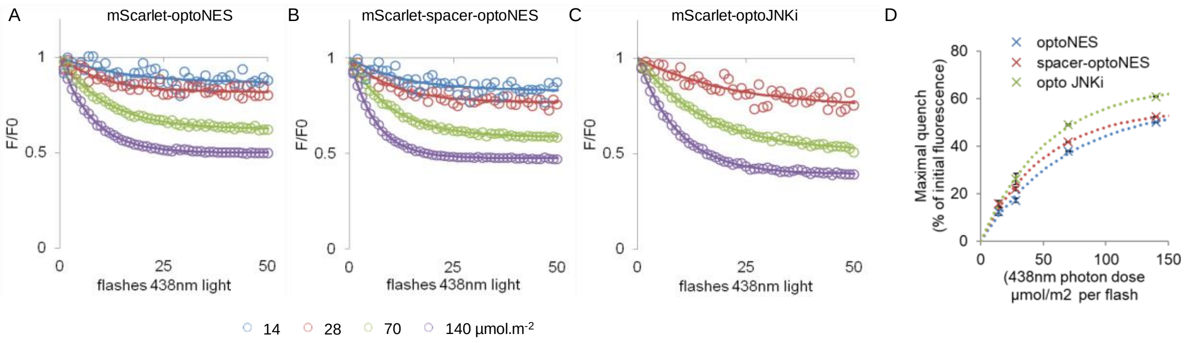

E

Actuator	*Quench limit %*	*sensitivity (µmol.m^{-2})*	ED$_{50}$ (µmol.m^{-2})	*relaxation time constant (s)*
Ypet-optoNES	52.9±0.3	715±10	42.3±1.2	4.2±0.1
Ypet-spacer-optoNES	30.8±1.8 ****	494±40 ****	13.4±4.5 ****	9.2±2.9 *
Ypet-optoJNKi	62.7±0.5****	1214±22 ****	46±2.2 (ns)	6.6±0.3 (n.s.)
mScarlet-optoNES	75±1.0	3498±279	290±31	3.0±0.1
mScarlet-spacer-optoNES	70±1.0 **	2016±95 ****	160±12 ***	3.2±0.1 (n.s.)
mScarlet-optoJNKi	81±1.0 **	4076±353 (n.s.)	260±32 (n.s.)	3.9±0.2 ****

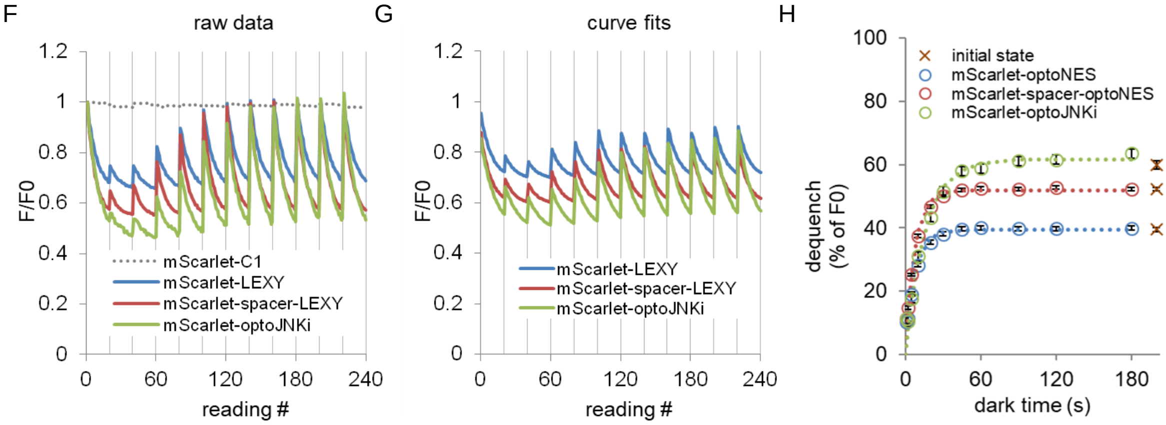

Supplementary Figure 13: Data for mScarlet tagged equivalent of Ypet-fused constructs shown in Fig. 6.

All conditions (n=3 wells) were the same except that fluorescence emission was detected via a blue-blocking 570nm long-pass filter. The best-fit values ± S.E. for Fig. 6A-C and S13A-C are compiled in the table in S13E. The best-fit values ± S.E. for Fig. 6E-G and S13F-H are compiled in Table 1. Source data are provided as a Source Data file.

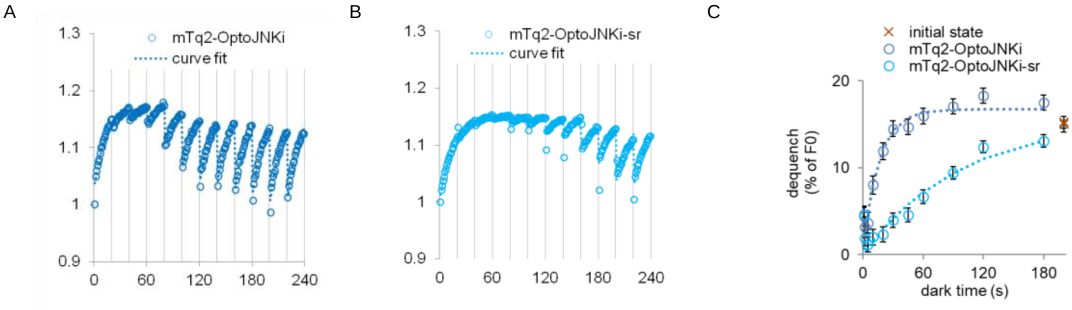

D

Actuator	relaxation time constant in dark (s)	ED_{50} (μmol.m^{-2})	Dequench limit (%)	Sensitivity (μmol.m^{-2})
mTq2-OptoJNKi	15.5±3.2	2.9±0.9	15.9±1.3	179±40
mTq2-OptoJNKi-sr	109±23	0.4±0.1	12.6±1.3	154±44

Supplementary Figure 14: Characterisation of mTq2-OptoJNKi-sr in comparison with mTq2-OptoJNKi.

The V416I mutant of LOV2 was inserted into OptoJNKi to generate OptoJNKi-sr (slow relaxing). As the kinetics of the JNK pathway are slow, this mutant was anticipated to respond sufficiently fast while requiring less frequent illumination to maintain the active state of this optogenetic actuator. This will help avoid phototoxicity while facilitating sample throughput in microscopy-based assays. The relaxation time constant was found to be ~7 times slower. The best-fit values ± S.E. (n=4 wells) are shown in C. Source data are provided as a Source Data file.

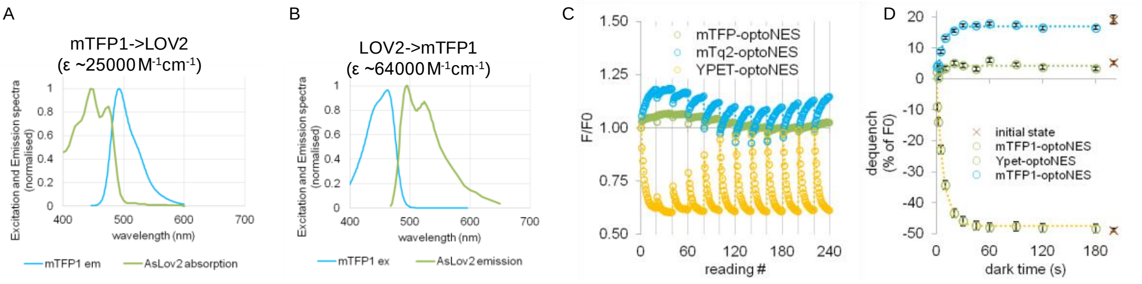

E

Fusion protein	Deqench limit (%)
mTurquoise2	20.2±1.3
Ypet	-51.1±0.7
mTFP1	4.9±0.7

Supplementary Figure 15: Spectral overlaps of normalised mTFP1 and *As*LOV2 spectra and the corresponding LOV2FRET dequench response.

(A-B) Overlaps exist for mTFP1 emission with AsLOV2 absorption spectra and vice versa, but they are much smaller than for mTq2, Ypet and mScarlet (Fig. 1). The overlap of normalised TFP1 emission and *As*LOV2 absorption spectra is greater, but considering that the extinction coefficient of *As*LOV2 is 2.6-fold lower than that of mTFP1, substantial unidirectional RET during linear illumination appears unlikely. (C) activation-relaxation cycle results were acquired as in Fig. 4, showing 438nm-induced dequenching of mTq2-optoNES, quenching of Ypet-optoNES as shown in earlier figures. mTFP1-optoNES shows only a minor level of dequenching behaviour, suggesting a relatively low level of resonance energy transfer from mTFP1 to *As*LOV2-445. (D) Relaxation of the dequenching/quenching values in darkness, obtained by curve fitting as described in the methods, suggests that in comparison with mTq2 and Ypet, mTFP1 exhibits minimal resonance energy transfer, consistent with the spectral comparisons (Figs. 1 and S10A-B). (E) Best-fit values ± S.E. for dequench (quench where <0) obtained from the data (n=3 wells) shown as averages in C, obtained by curve fitting as described in the methods. Source data are provided as a Source Data file.

Supplementary Table 1: Effects of mTq2, Ypet and mScarlet and the RET-reducing spacer between the fluorescent protein and the LOV2 domain

Fusion protein	Spacer between FP and LOV2	translocation ED_{50} (µmol 438nm photons.m^{-2})	Max. rate (% translocation per flash)	Initial Nuclear: Cytoplasmic Ratio
mTurquoise2	no	23.7±5.0	1.69±0.09	2.32±0.03
Ypet	no	171±29	3.19±0.22	1.76±0.03
mScarlet	no	151±13	6.14±0.21	3.78±0.05
mTurquoise2	yes	51.1±6.4	2.47±0.09	5.56±0.16
Ypet	yes	47.9±8.6	1.36±0.07	6.59±0.13
mScarlet	yes	50.3±6.3	2.98±0.11	9.93±0.15

Parameters shown are photon-sensitivity (ED_{50} or the photon flux per flash at the given flash rate evoking half-maximal reduction in % nuclear/cytoplasmic ratio) and max translocation rates, determined by fitting all data to a single-site model (dotted lines in Fig. 1), and initial nuclear:cytoplasmic ratio from the first image of the experiments (n=6 wells). The latter is calculated from average pixel intensities based on an eroded nuclear ROI and a cytoplasmic "band" ROI. The presence of low intensity pixels in the band ROI causes the initial ratio >1, but this nuclear bias will affect all samples equally. There was a significant difference in ED_{50} among the groups by F-test for individually expressed constructs without linker ($F=26.69(2,54)$, $P< 0.0001$). Source data are provided as a Source Data file.

Supplementary Table 2: Correlation analysis of the best-fit sensitivity and dequench limits for trimmed linker constructs

Correlation Matrix[1]

		Dequench	Sensitivity
Sensitivity	Pearson's r	-0.900 **	—
	p-value	0.003	—
	Spearman's rho	-0.857 *	—
	p-value	0.012	—
	Kendall's Tau B	-0.714 *	—
	p-value	0.015	—

Note. H_a is negative correlation

Note. * p < .05, ** p < .01, *** p < .001, one-tailed

[1]Data from Fig. 4E and Table 1A. Analysis was carried out using Jamovi (www.jamovi.org) with one-tailed F-test

Supplementary Table 3: AAV expression constructs used in this study

Table S3. AAV expression constructs used in this study.

Addgene ID	full plasmid name	ITRs	promoter	leader sequence	fluorescent tag	post-tag sequence	LOV2	Jalpha	C-term	Used in Fig
159941	AAV-synapsin-NLS-mTurquoise2-optoNES-WPRE	AAV2	human synapsin 1	NLS	mTurquoise2	short C1 MCS	wt 404-520	521-541	NES21 (LLKELADLNLD)	1,2,3,S5,S6,S10,S12,S15
159942	AAV-Synapsin-NLS-YPET-optoNES-WPRE	AAV2	human synapsin 1	NLS	Ypet	short C1 MCS	wt 404-520	521-541	NES21 (LLKELADLNLD)	1,6, S6, S12,S15
159943	AAV-Synapsin-NLS-mScarlet-optoNES-WPRE	AAV2	human synapsin 1	NLS	mScarlet	short C1 MCS	wt 404-520	521-541	NES21 (LLKELADLNLD)	1,S5,S6
159944	AAV-Synapsin-NLS-Dronpa-optoNES-WPRE	AAV2	human synapsin 1	none	Dronpa (22Gm3)	short C1 MCS	wt 404-520	521-541	NES21 (LLKELADLNLD)	1,S9
159945	AAV-Synapsin-NLS-mTFP1.0-optoNES-WPRE	AAV2	human synapsin 1	none	mTFP1.0	short C1 MCS	wt 404-520	521-541	NES21 (LLKELADLNLD)	S15
159946	AAV-Synapsin-mTurquoise2-3XNLS-optoNES-WPRE	AAV2	human synapsin 1	none	mTurquoise2	3xNLS and short MCS	wt 404-520	521-541	NES21 (LLKELADLNLD)	1,2,3, S8
159947	AAV-Synapsin-YPET-3XNLS-optoNES-WPRE	AAV2	human synapsin 1	none	Ypet	3xNLS and short MCS	wt 404-520	521-541	NES21 (LLKELADLNLD)	1,6, S8,S13
159948	AAV-Synapsin-mScarlet-3XNLS-optoNES-WPRE	AAV2	human synapsin 1	none	mScarlet	3xNLS and short MCS	wt 404-520	521-541	NES21 (LLKELADLNLD)	1, S8,S13
159949	AAV-Synapsin-mTurquoise-3XNLS-WPRE	AAV2	human synapsin 1	none	mTurquoise2	3xNLS and MCS	none	none	none	2,3
159950	AAV-Synapsin-Ypet-3XNLS-WPRE	AAV2	human synapsin 1	none	Ypet	3xNLS and MCS	none	none	none	7
159951	AAV-Synapsin-mCherry-3XNLS-WPRE	AAV2	human synapsin 1	none	mCherry	3xNLS and MCS	none	none	none	7
159952	AAV-Synapsin-miRFP670-3XNLS-WPRE	AAV2	human synapsin 1	none	miRFP670	3xNLS and MCS	none	none	none	1
159953	AAV-Synapsin-mTurquoise2-C1-WPRE	AAV2	human synapsin 1	none	mTurquoise2	C1 MCS	none	none	none	2,3,4,5,7,S10
159954	AAV-Synapsin-YPET-C1-WPRE	AAV2	human synapsin 1	none	Ypet	C1 MCS	none	none	none	6,8,S13
159955	AAV-Synapsin-mScarlet-C1-WPRE	AAV2	human synapsin 1	none	mScarlet	C1 MCS	none	none	none	S3,S10,S13
159956	AAV-Synapsin-mCherry-C1-WPRE	AAV2	human synapsin 1	none	mCherry	C1 MCS	none	none	none	S3
159957	AAV-Synapsin-NLS-mTurquoise2dC7-408LOV2-Ja(delta)-NES21-WPRE	AAV2	human synapsin 1	NLS	mTurquoise2 dC7	none	wt 408-520	521-541	NES21 (LLKELADLNLD)	4,S11,S12
159958	AAV-Synapsin-NLS-mTurquoise2dC8-408LOV2-Ja(delta)-NES21-WPRE	AAV2	human synapsin 1	NLS	mTurquoise2 dC8	none	wt 408-520	521-541	NES21 (LLKELADLNLD)	4,S11,S12
159959	AAV-Synapsin-NLS-mTurquoise2dC9-408LOV2-Ja(delta)-NES21-WPRE	AAV2	human synapsin 1	NLS	mTurquoise2 dC9	none	wt 408-520	521-541	NES21 (LLKELADLNLD)	4,S11,S12
159960	AAV-Synapsin-NLS-mTurquoise2dC10-408LOV2-Ja(delta)-NES21-WPRE	AAV2	human synapsin 1	NLS	mTurquoise2 dC10	none	wt 408-520	521-541	NES21 (LLKELADLNLD)	4,S11,S12
159961	AAV-Synapsin-NLS-mTurquoise2dC11-408LOV2-Ja(delta)-NES21-WPRE	AAV2	human synapsin 1	NLS	mTurquoise2 dC11	none	wt 408-520	521-541	NES21 (LLKELADLNLD)	4,S11,S12
159962	AAV-CMV-NLS-mTurquoise2-optoNES-WPRE	AAV2	CMV-IE	NLS	mTurquoise2	none	wt 408-520	521-541	NES21 (LLKELADLNLD)	4
159963	AAV-CMV-NLS-mTurquoise2dC7-408LOV2-Ja(delta)-NES21-WPRE	AAV2	CMV-IE	NLS	mTurquoise2 dC7	none	wt 408-520	521-541	NES21 (LLKELADLNLD)	4
159964	AAV-CMV-NLS-mTurquoise2dC10-408LOV2-Ja(delta)-NES21-WPRE	AAV2	CMV-IE	NLS	mTurquoise2 dC10	none	wt 408-520	521-541	NES21 (LLKELADLNLD)	4
159965	AAV-CMV-NLS-mTurquoise2dC11-408LOV2-Ja(delta)-NES21-WPRE	AAV2	CMV-IE	NLS	mTurquoise2 dC11	none	wt 408-520	521-541	NES21 (LLKELADLNLD)	4
159966	AAV-Synapsin-mTurquoise2-optoJNKi-WPRE	AAV2	human synapsin 1	NLS	mTurquoise2	short C1 MCS	wt 404-520	521-546	SR-JIP11	5,7,9,S12,S14
159967	AAV-Synapsin-Ypet-optoJNKi-WPRE	AAV2	human synapsin 1	NLS	Ypet	short C1 MCS	wt 404-520	521-546	SR-JIP11	6,7
159968	AAV-Synapsin-mScarlet-optoJNKi-WPRE	AAV2	human synapsin 1	NLS	mScarlet	short C1 MCS	wt 404-520	521-546	SR-JIP11	S10
159969	AAV-Synapsin-mTurquoise2-optoJNKi-C450A-WPRE	AAV2	human synapsin 1	NLS	mTurquoise2	short C1 MCS	404-520 C450A	521-546	SR-JIP11	5
159970	AAV-Synapsin-mTurquoise2-optoJNKi-V416I-WPRE	AAV2	human synapsin 1	NLS	mTurquoise2	short C1 MCS	404-520 V416I	521-546	SR-JIP11	9,S14
159971	AAV-Synapsin-mTurquoise2-optop38i3-WPRE	AAV2	human synapsin 1	NLS	mTurquoise2	short C1 MCS	wt 404-520	521-546	SR-MKK3DD3-13F	5,S12
159972	AAV-Synapsin-mTurquoise2-optop38i5-WPRE	AAV2	human synapsin 1	NLS	mTurquoise2	short C1 MCS	wt 404-520	521-546	SR-MKK3DD3-13F	5,S12
159973	AAV-Synapsin-NLS-YPET-optoNES.V416I-WPRE	AAV2	human synapsin 1	NLS	Ypet	short C1 MCS	404-520 V416I	521-541	NES21 (LLKELADLNLD)	8
159974	AAV-Synapsin-NLS-YPET-optoNES.I427V-WPRE	AAV2	human synapsin 1	NLS	Ypet	short C1 MCS	404-520 V416I	521-541	NES21 (LLKELADLNLD)	8
159975	AAV-Synapsin-NLS-YPET-optoNES.F434A-WPRE	AAV2	human synapsin 1	NLS	Ypet	short C1 MCS	404-520 V416I	521-541	NES21 (LLKELADLNLD)	8
159976	AAV-Synapsin- JNKKTR -miRFP670-WPRE	AAV2	human synapsin 1	JNKktr	iRFP670	C1 MCS	none	none	none	7,9

Supplementary References:

1. Andrews, David L. Chapter 14: Resonance Energy Transfer: Theoretical Foundations and Developing Applications in SPIE Digital Library Tutorials in Complex Photonic Media, Editor(s): Mikhail A. Noginov; Graeme Dewar; Martin W. McCall; Nikolay I. 1083 Zheludev Published: 2009
2. Lambert TJ. FPbase: a community-editable fluorescent protein database. Nat Methods. Apr;16(4):277-278. 2019
3. Gauden M, Crosson S, van Stokkum IHM, van Grondelle R, Moffat K, Kennis JTM. Low-temperature and time-resolved spectroscopic characterization of the LOV2 domain of Avena sativa phototropin 1. Proc. of SPIE Vol. 5463 97-104 2004
4. Diensthuber RP, Engelhard C, Lemke N, Gleichmann T, Ohlendorf R, Bittl R, Möglich A. Biophysical, mutational, and functional investigation of the chromophore-binding pocket of light-oxygen-voltage photoreceptors. ACS Synth Biol. Nov 21;3(11):811-9. 2014
5. Mastop M, Bindels DS, Shaner NC, Postma M, Gadella TWJ Jr, Goedhart J. Characterization of a spectrally diverse set of fluorescent proteins as FRET acceptors for mTurquoise2. Sci Rep. Sep 20;7(1):11999. 2017
6. Bindels DS, Haarbosch L, van Weeren L, Postma M, Wiese KE, Mastop M, Aumonier S, Gotthard G, Royant A, Hink MA, Gadella TW Jr. mScarlet: a bright monomeric red fluorescent protein for cellular imaging. Nat Methods. Jan;14(1):53-56. 2017
7. Niopek D, Wehler P, Roensch J, Eils R, Di Ventura B. Optogenetic control of nuclear protein export. Nat Commun. Feb 8;7:10624. 2016
8. Shaner NC, Steinbach PA, Tsien RY. A guide to choosing fluorescent proteins. Nat Methods. Dec;2(12):905-9. 2005
9. Sinnecker D, Voigt P, Hellwig N, Schaefer M. Reversible photobleaching of enhanced green fluorescent proteins. Biochemistry. May 10;44(18):7085-94. 2005
10. Ando R, Mizuno H, Miyawaki A. Regulated fast nucleocytoplasmic shuttling observed by reversible protein highlighting. Science. 2004;306(5700):1370-1373.
