# Supplementary material for: Resonance energy transfer sensitises and monitors in situ switching of LOV2-based optogenetic actuators
Source: Nat Commun. 2020 Oct 9;11:5107. doi: 10.1038/s41467-020-18816-8 (PMC7547724; doi:10.1038/s41467-020-18816-8)
Supplement: Supplementary file 9 — Description of Additional Supplementary Files [file 41467_2020_18816_MOESM9_ESM.pdf]

File name: Supplementary Movie 1

Description: Blue-light induces nuclear export of mTq2-optoNES in hippocampal neurons. See Supplementary Figure 5 for details.

File name: Supplementary Movie 2

Description: Blue-light induces nuclear export of YPET-optoNES in hippocampal neurons. Although this construct has a high initial cytoplasmic localisation, blue-light induced reduction of nuclear levels can still be observed, allowing quantification of light sensitivity. See Supplementary Figure 5 for details.

File name: Supplementary Movie 3

Description: Blue-light induces nuclear export of mScarlet-optoNES in hippocampal neurons. See Supplementary Figure 5 for details.

File name: Supplementary Movie 4

Description: Blue-light induces nuclear export of mTq2-spacer-optoNES in hippocampal neurons. See Supplementary Figure 5 for details.

File name: Supplementary Movie 5

Description: Blue-light induces nuclear export of YPET-spacer-optoNES in hippocampal neurons. See Supplementary Figure 5 for details.

File name: Supplementary Movie 6

Description: Blue-light induces nuclear export of mScarlet-spacer-optoNES in hippocampal neurons. See Supplementary Figure 5 for details.

File name: Supplementary Software

Description: This file includes a) ImageJ macro scripts used to quantify the responsiveness of optoNES constructs; b) An excel-based calculator to predict the dynamic adduct state of a LOV2 switch exposed to a defined illumination pattern, based on switch-specific parameters measured as described in the text.
