# Supplementary material for: Resonance energy transfer sensitises and monitors in situ switching of LOV2-based optogenetic actuators
Source: Nat Commun. 2020 Oct 9;11:5107. doi: 10.1038/s41467-020-18816-8 (PMC7547724; doi:10.1038/s41467-020-18816-8)
Supplement: Supplementary file 10 — Reporting Summary [file 41467_2020_18816_MOESM10_ESM.pdf]

nature research

Corresponding author(s): Courtney MJ

Last updated by author(s): Aug 31, 2020

Reporting Summary

Nature Research wishes to improve the reproducibility of the work that we publish. This form provides structure for consistency and transparency in reporting. For further information on Nature Research policies, see our Editorial Policies and the Editorial Policy Checklist.

Statistics

For all statistical analyses, confirm that the following items are present in the figure legend, table legend, main text, or Methods section.

n/a	Confirmed	
☐	☒	The exact sample size (n) for each experimental group/condition, given as a discrete number and unit of measurement
☐	☒	A statement on whether measurements were taken from distinct samples or whether the same sample was measured repeatedly
☐	☒	The statistical test(s) used AND whether they are one- or two-sided<br>*Only common tests should be described solely by name; describe more complex techniques in the Methods section.*
☒	☐	A description of all covariates tested
☐	☒	A description of any assumptions or corrections, such as tests of normality and adjustment for multiple comparisons
☐	☒	A full description of the statistical parameters including central tendency (e.g. means) or other basic estimates (e.g. regression coefficient) AND variation (e.g. standard deviation) or associated estimates of uncertainty (e.g. confidence intervals)
☐	☒	For null hypothesis testing, the test statistic (e.g. F, t, r) with confidence intervals, effect sizes, degrees of freedom and P value noted<br>*Give P values as exact values whenever suitable.*
☒	☐	For Bayesian analysis, information on the choice of priors and Markov chain Monte Carlo settings
☒	☐	For hierarchical and complex designs, identification of the appropriate level for tests and full reporting of outcomes
☐	☒	Estimates of effect sizes (e.g. Cohen's d, Pearson's r), indicating how they were calculated

Our web collection on statistics for biologists contains articles on many of the points above.

Software and code

Policy information about availability of computer code

Data collection	Imaging data collected by Attovision 1.6 software provided with the Beckton Dickinson Pathway 855 High-Content Analyzer<br>Plate reader data collected with BMG Optima software version 2.20R2
Data analysis	Image analysis was performed using ImageJ version 1.52n and above, using the attached scripts;<br>Curve fitting and statistical analysis were performed with GraphPad prism version 7 and 8 after collation of data with MS Excel versions2012 and 2016; Jamovi 1.1.7.0 was also used where indicated.<br>Adduct fraction predictions were carried out with the attached custom Excel-based calculator.

For manuscripts utilizing custom algorithms or software that are central to the research but not yet described in published literature, software must be made available to editors and reviewers. We strongly encourage code deposition in a community repository (e.g. GitHub). See the Nature Research guidelines for submitting code & software for further information.

Data

Policy information about availability of data

All manuscripts must include a data availability statement. This statement should provide the following information, where applicable:

- Accession codes, unique identifiers, or web links for publicly available datasets
- A list of figures that have associated raw data
- A description of any restrictions on data availability

Source data are provided with this paper. Figure data are in source data files. Raw imaging data, together with associated metadata files, for figures 1-9 are available at reserve doi 10.5281/zenodo.3882572. Scripts used for controlling the image acquisition are within this metadata. All data in this manuscript are available from the corresponding author upon request. All plasmids used in this manuscript were new plasmids and deposited in addgene under IDs 159941-159976. They are described in detail in Supplementary Table 3. These plasmids were generated in part with the help of previously generated plasmids (including addgene IDs 89744, 89749, 127862) and plasmids provided by other labs (including addgene IDs 26019, 59148). The FBbase database was also used for this work (https://www.fpbase.org/).

Field-specific reporting

Please select the one below that is the best fit for your research. If you are not sure, read the appropriate sections before making your selection.

☒ Life sciences ☐ Behavioural & social sciences ☐ Ecological, evolutionary & environmental sciences

For a reference copy of the document with all sections, see nature.com/documents/nr-reporting-summary-flat.pdf

Life sciences study design

All studies must disclose on these points even when the disclosure is negative.

Sample size	In all cases a minimum of n= 3 distinct replicates was used. This number was chosen because it is typically considered to be the minimum for statistical analysis, and is specified in the editorial requirements to be a minimum. Where additional sources of noise are expected, such as the nuclear translocation measurements where cell functionality is important in primary cultures where cells are more heterogeneous than a cell line like 293 cells, double the number of replicates (or at least 4) was used to mitigate this risk. Also the initial mTq2-optoNES v mTq2-spacer-optoNES comparison (Fig. 2) was carried out at n=6 because the noise level was not initially clear; in the latter case one replicate did not reach threshold and therefore only 5 replicates could be included
Data exclusions	We stated in the methods, "In the case of the curve-fitting of the data in Fig. 2C-D the first of the 50 datapoints, was discarded due to an evident systematic artefact affecting most replicates, probably related to the imperfect correction to the control in this case (Supplementary Figure 10A). No other data points were discarded in this or any other experiment."
Replication	Experiments were carried out on the basis of smaller pilot experiments to determine trends and the number of reading cycles required for the experiments shown. All pilot findings were replicated. The number of times each experiment was replicated under specified identical conditions is shown in each figure legends
Randomization	no covariates were measured. Cells were allocated at random into experimental wells from large-scale pools, at ~10000 per well (depending on the cell type, see methods for specific numbers)
Blinding	Data acquisition and analysis were automated using scripts.

Reporting for specific materials, systems and methods

We require information from authors about some types of materials, experimental systems and methods used in many studies. Here, indicate whether each material, system or method listed is relevant to your study. If you are not sure if a list item applies to your research, read the appropriate section before selecting a response.

Materials & experimental systems

n/a	Involved in the study
☒	☐ Antibodies
☐	☒ Eukaryotic cell lines
☒	☐ Palaeontology and archaeology
☒	☐ Animals and other organisms
☒	☐ Human research participants
☒	☐ Clinical data
☒	☐ Dual use research of concern

Methods

n/a	Involved in the study
☒	☐ ChIP-seq
☒	☐ Flow cytometry
☒	☐ MRI-based neuroimaging

Eukaryotic cell lines

Policy information about cell lines

Cell line source(s)	HEK293 from ATCC
Authentication	None
Mycoplasma contamination	HEK293 tested for mycoplasma by Hoechst staining and found to be negative for signs of mycoplasma.
Commonly misidentified lines (See ICLAC register)	The ICLAC register mentions HeLa as a potential contaminant of 293. Our study required an easy to transfect line and one that would generate AAV particles. HeLa cells would also have been suitable for the former but would not have been suitable for the latter (because we did not use Adenovirus helper), in contrast to the 293 cells we used.
